# Clustering of Tissue-Specific Sub-TADs Accompanies the Regulation of *HoxA* Genes in Developing Limbs

**DOI:** 10.1371/journal.pgen.1004018

**Published:** 2013-12-26

**Authors:** Soizik Berlivet, Denis Paquette, Annie Dumouchel, David Langlais, Josée Dostie, Marie Kmita

**Affiliations:** 1 Unité de génétique et développement, Institut de recherches cliniques de Montréal, Montréal, Québec, Canada; 2 Department of Biochemistry and Goodman Cancer Research Center, McGill University, Montréal, Québec, Canada; 3 Unité de génétique moléculaire, Institut de recherches cliniques de Montréal, Montréal, Québec, Canada; 4 Department of Medicine, University of Montréal, Montréal, Québec, Canada; Ludwig Institute for Cancer Research, University of California San Diego, United States of America

## Abstract

*HoxA* genes exhibit central roles during development and causal mutations have been found in several human syndromes including limb malformation. Despite their importance, information on how these genes are regulated is lacking. Here, we report on the first identification of *bona fide* transcriptional enhancers controlling *HoxA* genes in developing limbs and show that these enhancers are grouped into distinct topological domains at the sub-megabase scale (sub-TADs). We provide evidence that target genes and regulatory elements physically interact with each other through contacts between sub-TADs rather than by the formation of discreet “DNA loops”. Interestingly, there is no obvious relationship between the functional domains of the enhancers within the limb and how they are partitioned among the topological domains, suggesting that sub-TAD formation does not rely on enhancer activity. Moreover, we show that suppressing the transcriptional activity of enhancers does not abrogate their contacts with *HoxA* genes. Based on these data, we propose a model whereby chromatin architecture defines the functional landscapes of enhancers. From an evolutionary standpoint, our data points to the convergent evolution of *HoxA* and *HoxD* regulation in the fin-to-limb transition, one of the major morphological innovations in vertebrates.

## Author Summary

*Hox* genes encode transcription factors with crucial roles during development. These genes are grouped in four different clusters names *HoxA*, *B*, *C*, and *D*. Mutations in genes of the *HoxA* and *D* clusters have been found in several human syndromes, affecting in some cases limb development. Despite their essential role and contrary to the genes of the *HoxD* cluster, little is known about how the *HoxA* genes are regulated. Here, we identified a large set of regulatory elements controlling *HoxA* genes during limb development. By studying spatial chromatin organization at the *HoxA* region, we found that the regulatory elements are spatially clustered regardless of their activity. Clustering of enhancers define tissue-specific chromatin domains that interact specifically with each other and with active genes in the limb. Our findings give support to the emerging concept that chromatin architecture defines the functional properties of genomes. Additionally, our study suggests a common constraint of the chromatin topology in the evolution of *HoxA* and *HoxD* regulation in the emergence of the hand/foot, which is one of the major morphological innovations in vertebrates.

## Introduction

The *Hox* gene family encodes transcription factors with central roles in patterning of the body plan and organogenesis. *Hox* genes are grouped into clusters in most animal species, and mammals possess 39 genes divided into four clusters named *HoxA* to *HoxD*. In mice, deletion of the *HoxA* cluster is embryonic lethal [1]–[2] whereas mutants lacking *HoxB*, *HoxC*, or *HoxD* are viable at least until birth [3]–[5]. Inactivation of individual *Hox* genes identified *Hoxa13* as a gene required for proper placenta function and thus embryonic survival [2], [6]–[7]. Mutations in *HoxA* genes have been found in various human syndromes (e.g. HFGS-OMIM140000, Guttmacher syndrome-OMIM176305, MRKH-OMIM277000) including limb malformations. Studies of gene inactivation in mice demonstrated that genes located at the 5′ end of the *HoxA* cluster (*Hoxa9*–*13*) are required for proper patterning of the three limb segments: the upper arm (humerus; *Hoxa9*, *10*), lower arm (radius and ulna; *Hoxa10, 11*), and the hand/foot (autopod; *Hoxa13*) [6], [8]–[11].

Despite their pivotal roles during embryogenesis, little is known about the regulation of *HoxA* genes. This is in contrast to *HoxD*, which transcriptional control has been more thoroughly studied, especially in the limb where the *HoxD* genes play partially overlapping functions with *HoxA* [12]. Expression at the *HoxA* and *D* clusters follows similar dynamics during limb development, and occurs in two phases [12]. In the first phase, which starts at embryonic day 9.5 of development (E9.5), expression at both clusters is comparable suggesting that the control mechanisms are likely similar. During this phase, gene expression generally follows the collinear strategy observed in the trunk, characterized by sequential gene activation from one end of the cluster (*Hox1*) to the other (*Hox13*), with early activated genes expressed throughout the limb bud and those activated later (*Hox10-13*) gradually restricted to posterior cells [13]. In contrast, the expression domains of *HoxA* and *HoxD* genes partly differ in the second phase (from E11.5 onwards), suggesting some differences in the regulatory mechanisms controlling the clusters in this later phase.

Previous studies show that transcription at the *HoxD* cluster is regulated long-distance by enhancers in several tissues (reviewed in [14]). Notably, expression of *Hoxd10* to *Hoxd13* in the distal part of the limb bud (presumptive hand/foot) is controlled by several remote *cis*-regulatory sequences located in the gene desert upstream of the cluster [15]. Hands/feet, in particular digits, are evolutionary novel structures and the hallmark of Vertebrate adaptation to terrestrial habitats. The fact that *Hoxa10* and *Hoxa13* are also expressed in the presumptive hand/foot domain therefore raised the possibility that specific recruitment of *HoxA* and *HoxD* gene functions in developing digits stem from the implementation of similar *cis*-regulatory elements during the fin-to-limb transition. Whereas sequence conservation analysis of the region upstream of these clusters did not identify cognate *cis*-regulatory elements driving *HoxA* expression in limbs [16], BAC transgenesis revealed the existence of a “digit” enhancer activity located between 250 and 500 kb upstream of the *Hoxa13* gene, in the neighborhood of the 3-hydroxyisobutyrate dehydrogenase (*Hibadh*) gene [17]. As *Hibadh* is also expressed in distal limb buds [16], this study could not resolve whether the “digit” enhancer activity detected within that region controls *Hibadh*, *Hoxa10/13*, or both. Thus, the enhancer sequence(s) and whether *HoxA* expression in limbs is regulated by long-range control mechanisms has remained unknown.

It was previously shown that control DNA elements could regulate the expression of remote genes by physically interacting with them [18]. Physical contacts between chromatin segments can be measured using the chromosome conformation capture (3C) methods, a series of assays that use proximity-based ligation to infer the three-dimensional organization of genomes [19]. 3C assays were used to show that regulation of *HoxD* genes in the presumptive digit domain is mediated by physical contacts with remote enhancers, and led to a model whereby expression of *Hoxd10* to *d13* associates with the formation of DNA loops between the genes and regulatory islands [15]. This was further supported by Fluorescence In Situ Hybridization data showing the co-localization of *HoxD* genes and one of its regulatory islands, specifically in digit progenitor cells [20]. Whether the proximity between target genes and regulatory DNA elements requires transcription appears to be loci-dependent and it remains unknown whether a given mechanism prevails over others. Indeed, while such “loops” were sometimes reported in absence of transcription [21]–[22], transcription factors requirement for DNA looping was uncovered at the *β*-globin locus [23]–[24] and *Igh* gene [25].

Here, we show that during limb development, expression of *HoxA* genes is controlled by multiple remote enhancers located upstream of the cluster. In limb cells, these enhancers are grouped into distinct sub-megabase topological domains (sub-TADs) that contact each other and the sub-TADs containing target genes. In head tissues, the topology is drastically different, modifying both gene-enhancer and enhancer-enhancer interactions. Interestingly, enhancers located in the same sub-TAD are active in distinct subset of limb cells suggesting that spatial clustering of enhancers does not simply reflect enhancer co-activity. We also present evidence that enhancer-*HoxA* contacts are maintained even when enhancer activity is suppressed, suggesting that the *HoxA* regulatory region acquires a permissive conformation prior to gene activation. We suggest a model whereby sub-TAD formation and/or contacts between sub-TADs define the *cis*-regulatory network controlling gene expression. From an evolutionary perspective, this first extensive characterization of *HoxA* regulation in developing limbs provides new insights into the evolution of *Hox* regulation in the emergence of hand/foot. Our study suggests that while the DNA sequences of the distal limb enhancers for *HoxA* and *HoxD* genes are different and have likely emerged independently, chromosome partitioning into topological domains has similarly constrained the evolution of *HoxA* and *HoxD* cis-regulatory landscapes underlying the emergence of digits, one of the major morphological innovations in Vertebrates.

## Results

### Multiple candidate limb enhancers are located upstream of the *HoxA* cluster

**Figure 1:**
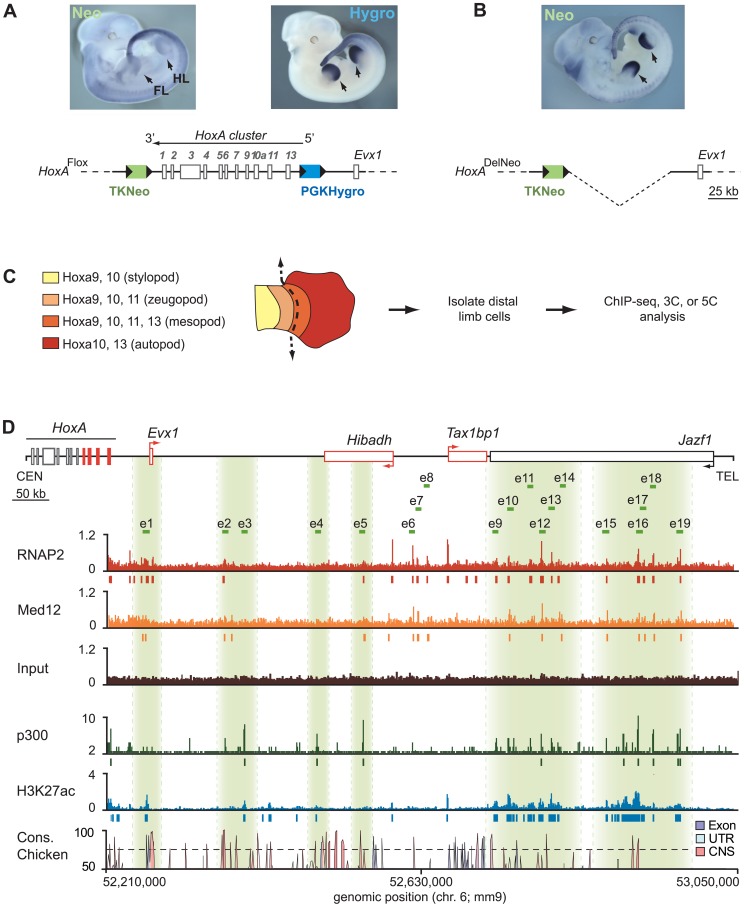
Candidate limb enhancers reside on the telomeric side of the *HoxA* cluster. **A, B.** Distal limb enhancer activity lies upstream of the *HoxA* cluster and does not require sequences within it. Expression of the Neomycin and Hygromycin reporter genes flanking the cluster were analyzed by whole mount *in situ* hybridization on E11.5 embryos. In embryos where the *HoxA* cluster is intact (**A**), expression of the upstream Hygromycin reporter was detected in the distal part of the limb while downstream neomycin transcripts were not. TKNeo: minimal thymidine kinase promoter upstream of Neomycin reporter gene. PGKHygro: minimal phosphoglycerate kinase-1 promoter upstream of Hygromycin reporter gene. Arrow above the *HoxA* cluster diagram shows transcription direction. **B.** Neomycin expression after deletion of the cluster and PGKHygro by recombination of *loxP* sites flanking the reporter genes shows that sequences within the cluster are not required for distal limb enhancer activity. **C.** Distal limb cells analyzed in this study express 5′ *HoxA* genes (*Hoxa9* to *a13*). *HoxA* gene expression in developing limbs is illustrated on the *left*. The dotted line indicates the area micro-dissected to collect distal limb cells for analysis. Stylopod: upper arm, zeugopod: lower arm, mesopod: wrist, autopod: hand. **D.** The position of candidate enhancer sequences was identified by ChIP-seq. Proteins known as being enriched at active enhancers (RNAP2, Med12, p300) and the H3K27Ac histone mark was examined as described in Materials and Methods. The y-axis corresponds to “reads per million” except for the p300 data where the number of sequence reads is shown. Colored rectangles below each track indicate the position of significant peaks. The position of candidate enhancers (e1 to e19) is highlighted in green below the genomic region characterized, where transcriptionally active genes are in red and arrows indicate transcription direction. Sequence conservation in the chicken is shown on the bottom.

To identify enhancer sequences regulating *HoxA* expression during limb development, we used a combination of genetic and genomics approaches that probe enhancer features in mouse embryos. We first tested whether *HoxA* transcription in developing limbs involves *cis*-regulatory sequences outside of the gene cluster itself. To this end, we used two mutant lines with targeted genomic rearrangements at the *HoxA* cluster [1]–[2] to monitor activation of reporter transgenes by surrounding enhancer activity (Figure 1A,B). Whole mount *in situ* hybridization shows that a neomycin reporter transgene located downstream of *Hoxa1* is not expressed in limbs at E11.5 (Figure 1A, *left*). In contrast, we found that a hygromycin transgene inserted at the opposite end of the cluster, 3.5 kb upstream of *Hoxa13* is robustly transcribed in distal limbs at this stage (Figure 1A, *right*). These expression patterns correlate well with the expression profile of the endogenous *HoxA* genes adjacent to the reporter transgenes. Upon deletion of the entire *HoxA* cluster, the neomycin transgene becomes activated in distal limbs suggesting that sequences upstream of the cluster are sufficient to trigger distal expression (Figure 1B). These results support the previously proposed hypothesis that a 250 kb region in the neighborhood of *Hibadh* contains an enhancer activity controlling *HoxA* expression in developing limbs [17].

Given the results described above, we focused our analysis on the genomic region upstream of the cluster. To identify active enhancers in distal limbs, we used dissected distal forelimbs (Figure 1C), which are composed of cells expressing mainly *Hoxa10* and *a13*, but also a small amount of *Hoxa9* and *a11* from the presumptive wrist domain (mesopod). Active enhancers are characterized by the binding of several proteins including RNA polymerase II (RNAP2), and subunits of Mediator like Med12 [26]. We therefore mapped candidate enhancer sequences by identifying genomic sites enriched in these proteins using chromatin immunoprecipitation combined with deep sequencing (ChIP-seq) in cells isolated from E12.5 distal limb buds (Figure 1D). This data was considered together with previously published datasets derived from whole limb buds for the transcriptional co-activator p300 [27] and acetylated histone H3 lysine 27 (H3K27Ac), which also mark active enhancers [28]. Sequences distinct from proximal promoters (RefSeq) that were bound by RNAP2 and at least one other mark, or by both p300 and H3K27Ac were retained as candidate enhancers. Using these criteria, 19 putative enhancers were identified within 850 kb upstream of *Hoxa13* (Figure 1D, *top*).

### *HoxA* expression in developing limbs relies on several enhancer elements

**Figure 2:**
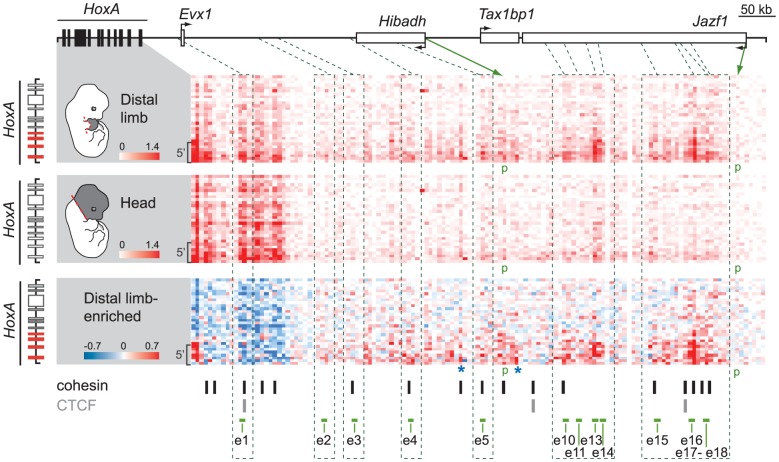
Several candidate enhancers interact specifically with 5′ *HoxA* genes in the limb. Physical contacts between the *HoxA* cluster and the upstream genomic region containing candidate enhancers were measured by 5C-seq in distal limb (*top*) and the head (*middle*) of E12.5 embryos. 5C data is represented in heatmap form with the color intensity of each pixel reflecting the frequency of interaction between two genomic regions. Contact frequency is according to the respective color scales and corresponds to the number of sequence reads. Most predicted enhancers (4;5;10;11;13;14;15;16;17;18) interacted long-distance specifically with 5′ *HoxA* genes and these interactions were enriched in the limb (*bottom* panel). Color scale in the bottom panel contrasts interactions enriched in the limb (red) and in the head (blue). Green dotted lines link the position of enhancers along the genomic region to the corresponding 5C fragments. Brackets on the left hand side of each heatmap show the area containing *Hoxa9*, *a10*, *a11*, and *a13*. Green arrows point to the chromatin fragments containing the *Hibadh* and *Jazf1* promoters (p). Blue stars highlight other limb-enriched interactions with *HoxA* genes that do not correspond to candidate enhancers. Restriction fragments corresponding to enhancer e6–8, 12, and 19 could not be included in the 5C design as they fell into regions that were not amenable to 5C (see Materials and Methods). Loci bound by cohesin (black bars) and/or CTCF (grey bars) in limb bud cells at E11.5 [35] are indicated below the 5C heatmaps. Note that most interactions identified by 5C correspond to loci bound by cohesin.

The number of candidate sequences identified upstream of *HoxA* was rather large, particularly compared to *HoxD* for which seven enhancers have been identified [15]. Also, in contrast to the gene desert surrounding *HoxD*, the region upstream of *HoxA* encompasses several genes (Figure 1D). Candidate *HoxA* enhancers therefore reside amidst other genes including *Hibadh*, *Tax1bp1*, and *Jazf1*, for which expression has been reported in the limb [16]. As ChIP-seq datasets cannot resolve the targets of enhancers, we used a structural approach to assess the potential interactions of the candidate enhancers with *HoxA* genes. We profiled the interaction pattern of the *HoxA* cluster with the upstream 850 Kb region in distal limb buds and head tissues using 5C technology combined with deep sequencing [29]–[30], which provides insights into chromatin architecture at high resolution (down to 4–6 kbs on average). We found that the 5′ part of *HoxA*, containing *Hoxa9* to *Hoxa13*, frequently interacts with several regions upstream of the cluster (Figure 2, *top*, Figure S1), and that most of these regions contain the candidate limb enhancers (Figure 2, *bottom*). In contrast, none of the enhancers interact with the 3′ part of the cluster containing *Hoxa1* to *Hoxa7* (Figure 2), which have no detectable expression in limb buds at this developmental stage. This result is reminiscent of the distal enhancers controlling the 5′ *HoxD* genes, which are also located upstream of the cluster and specifically interact with genes located in the 5′ part [15]. Interestingly, previous studies based on Hi-C analysis revealed that the *HoxA* and *HoxD* clusters each span a junction between two so-called “topologically associated domains” (TADs), with 3′ genes residing into one TAD and the 5′ part extending into the other [31]. TADs are thought to represent a basic unit of chromatin organization at the megabase-scale that is largely conserved between cell types [32]. Our data therefore points to a common relationship between chromatin topology and the limb regulatory landscapes of the *HoxA* and *HoxD* clusters.

Interestingly, sequences with the highest interaction frequencies with 5′*HoxA* genes (e10, 13, 14 and e15, 16, 18) locate farther from the cluster, within the *Jazf1* gene, and correspond to those loci most enriched in marks typical of active enhancers (Figure 1D). High interaction frequencies being associated either with stronger, more abundant and/or stable spatial contacts, these data likely reflect a prevalent activity of these enhancers in distal limbs. In contrast to the other enhancers, e1 and e3 do not show enriched interactions with the 5′ part of the *HoxA* cluster in distal limbs compared to head tissue (Figure 2, *bottom*). e1 is located close to *Evx1*, within a region of high interaction frequencies with *HoxA* both in limb and head tissues. This is not the case for e3 so we further tested interaction frequencies between *Hoxa13* and e2 to e5 using 3C (Figure S2). This analysis shows higher frequency of interactions between these enhancers and *Hoxa13* specifically in the limb. Yet, based on our 5C data, these interactions are modest compared to those observed for the other enhancers (Figure 2). Interestingly, contacts such as those with e5, e13 and e15 were also observed in the head at low frequencies (Figure 2). As there is no evidence of *HoxA* expression in the head at the stage analyzed, the contacts observed might be evidence of default chromatin architecture in this tissue. Alternatively, these enhancers may drive *HoxA* expression in head tissues at levels below detection by whole-mount *in situ* hybridization. Finally, our 5C data also reveals high interaction frequencies with at least two loci that have no apparent characteristics of transcriptional enhancers (Figure 2 bottom, blue stars). These may reflect additional structural anchors that stabilize the chromatin architecture, such as those mediated by CTCF and Cohesin [33]–[34]. Interestingly, loci bound either by CTCF or cohesin in limb buds have been recently identified [35] and comparison with our data shows that almost all loci interacting with 5′ *HoxA* genes overlap with either CTCF or cohesin binding (Figure 2, *bottom*).

**Figure 3:**
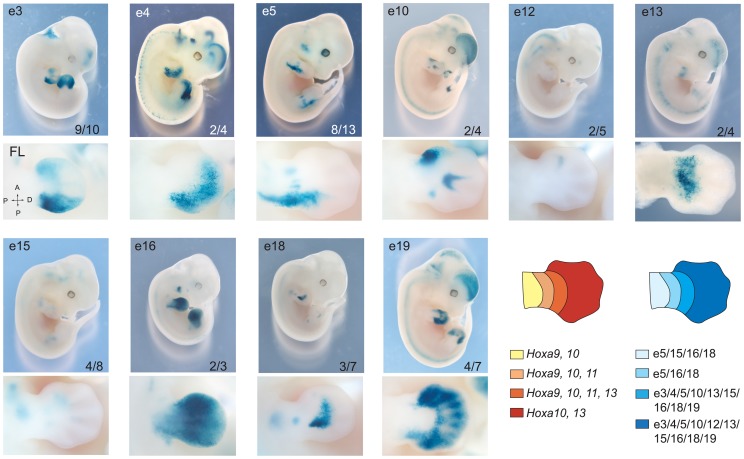
Overlapping domain-specific enhancer activity regulates 5′ *HoxA* genes in distal limbs. Transgenic analysis of enhancer candidates. In each row, top panels show LacZ staining in whole embryos and higher magnification of the limb bud is shown below. Tested enhancers are indicated at the top of each panel, and the number at the bottom represents embryos positive for the pattern reported over the total number of transgenic specimens analyzed. Lower panels present a dorsal view of corresponding forelimbs (FL) except for e13, which is shown ventrally. Diagrams on the right summarize the expression patterns of *HoxA* genes (*left*), and the activity of each enhancer (*right*) in the developing limb at E12.5, respectively.

Having confirmed the spatial proximity between 5′ *HoxA* genes and most of our candidate enhancers, we proceeded to test their *in vivo* activity in the mouse by transgenesis. Putative enhancer sequences were subcloned into vectors carrying the *β*-globin minimal promoter and *lacZ* reporter. Except for e1 and e2, X-Gal staining in transgenic embryos shows that all candidate enhancers tested activates transcription in developing limbs (Figure 3, Table S1). Interestingly, the confirmed enhancers exhibit distinct but overlapping activity domains in limb buds, and all trigger expression in the presumptive hand/foot (Figure 3). While some are active mostly in the distal part of the limb (e3, 4, 5, 10, 12, 13), others are functional also in the proximal domain (e5, 16, 18). The only candidate enhancers that fail to trigger reporter expression in our transgenic assays are e1 and e2 (Table S1). The absence of activity for these two candidates indicates either that these sequences are not limb enhancers or that the transgenes did not include all the necessary sequences to reflect their transcriptional activities. For e1, our 5C data (Figure 2) neither supports nor disagrees with it being a *HoxA* enhancer since it lies within a large region of high interaction frequency. Interestingly, e1 is located within a 50 kb DNA fragment that was previously shown to trigger gene expression in distal limbs [2], suggesting that it is possibly a *bona fide* limb enhancer but that some sequences required for its activity are absent from the 2.9 kb fragment tested in our transgenic assay. Similarly, absence of X-Gal staining in e2 transgenic embryos does not prove that e2 is not an enhancer. Yet, the fact that it does not strongly interact with 5′ *HoxA* genes in our 3C and 5C assays suggest that e2 may not be tightly linked to the regulation of *HoxA* genes. Nonetheless, analysis of the other identified enhancers shows that multiple enhancers with overlapping domain-specific activities regulate transcription at the *HoxA* cluster in the limb.

### *HoxA*-enhancer contacts do not require enhancer activity

While “DNA looping” is associated with long-range transcriptional control, the extent to which spatial structure exists prior to or as a consequence of enhancer activation remains elusive. This issue partly originates from the fact that most studies have compared the spatial distance of enhancers and target gene(s) in tissues expressing the genes with others where they are never expressed. To gain insight into the causative relationship between spatial proximity and long-distance enhancer regulation, we examined the outcome of enhancer silencing on long-range enhancer-gene interactions in developing limbs. During limb development, the transcriptional repressor Gli3R negatively regulates the expression of *HoxA* genes [36]–[37]. While *Gli3* is expressed almost throughout the limb in wild type (wt) embryos, the Gli3R domain is restricted anteriorly as a consequence of posterior Sonic hedgehog (Shh) signaling emanating from the Zone of Polarizing Activity (ZPA), which blocks processing of the full length Gli3 protein into its truncated repressor form [38]. In *Shh*−/− limbs, the Gli3R functional domain extends posteriorly leading to the down-regulation of *HoxA* as well as *HoxD* genes [36]–[37].

**Figure 4:**
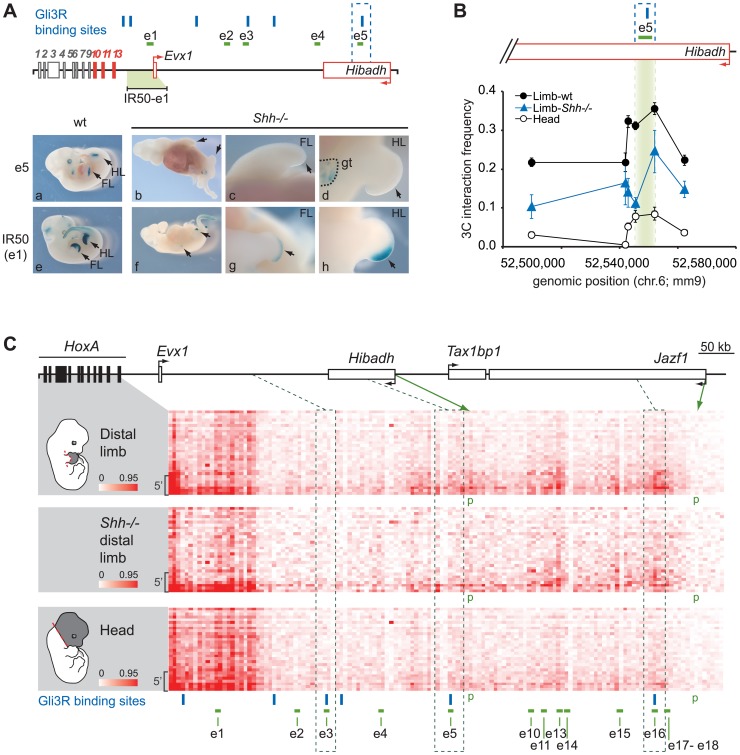
Regulatory *HoxA* contacts are independent of enhancer activity. **A.** Analysis of e1 and e5 activity in *Shh−/−* limbs. Upper panel: Scheme representing loci bound by Gli3R (blue bars) along the *HoxA* regulatory region. The IR50 transgene used as control contains the 50 kb intergenic region to *Hoxa13* and *Evx1*, which includes e1. Active genes are shown in red, and arrows indicate the position of promoters and transcription direction. Lower panel: LacZ staining showing e5 (a–d) and e1 (e–h) transcriptional activity, in wt (a; e) and *Shh−/−* embryos (b–d; f–h). **B.** Long-range e5 interaction with *Hoxa13* is independent of its activity. The physical proximity between the *Hoxa13* gene and e5 was measured by 3C. The position of e5 is highlighted in green. Interaction frequency were measured compared to a BAC 3C library as described in the Materials and Methods. Error bars correspond to the standard error of the mean. **C.** Physical contacts between the *HoxA* cluster and the upstream genomic region measured by 5C-seq in wild-type distal limb (*top*), *Shh−/−* mutant distal limb (*middle*), and the head (*bottom*) of mouse embryos. 5C data is presented in the form of heatmaps according to color scales as described in Figure 2. The limb-specific interaction pattern between enhancers and the 5′ *HoxA* genes are similar in *Shh−/−* (*middle* panel) and wt distal limb buds (*top* panel) albeit with some interaction frequencies slightly reduced. Dotted lines delineate the regions containing the enhancers bound by Gli3R (e3, e5 and e16). Gli3R sites are represented with blue bars. Brackets on the left hand side of each heatmap show the area containing *Hoxa9*, *a10*, *a11*, and *a13*. Green arrows indicate the chromatin fragments containing the *Hibadh* and *Jazf1* promoters (p). Restriction fragments corresponding to enhancer e6–8, 12, and 19 are not shown in the heatmaps as they fell into regions that were not amenable to 5C (see Materials and Methods).

Amongst the *HoxA*-associated limb enhancers identified, we found several that overlap with loci bound by Gli3R in the limb (e3, e5, e9 and e16; [39]). The activity of these enhancers should thus be suppressed in *Shh−/−* mutant. Of these, e5 is of particular interest because it triggers robust gene expression (Figure 3), and there is no other limb enhancer in its genomic neighborhood allowing us to assess its interaction frequency with *HoxA* without potential interference from surrounding enhancers. We first verified the activity of e5 in *Shh*−/− by generating mutant embryos homozygous for *Shh* inactivation and carrying the e5 transgene. X-Gal staining shows that e5 activity is suppressed in limbs upon inactivation of *Shh* (Figure 4A *a–d*, compare *a* to *b*) while still functional in the developing genitalia (Figure 4A, *panel d*). In contrast, a transgene containing the e1 enhancer, which does not overlap with a Gli-bound locus, remained expressed in a *Shh*−/− background (Figure 4A *f–h*) although in a smaller domain consistent with *Shh−/−*embryos having reduced limb size ([40]; Figure 4A, compare *e* to *f*).

To assess whether *HoxA*-enhancer proximity requires enhancer activity, we measured contacts between *Hoxa13* and e5 in wt and *Shh*−/− distal limb buds from E11.5 embryos. As e5 activity is suppressed in the absence of *Shh*, the enhancer should no longer interact with *Hoxa13* if enhancer activity is required for the contact. 3C analysis shows that e5 interacts with *Hoxa13* even in the absence of *Shh* (Figure 4B). Although interaction frequencies are lower than in wt limbs, the interaction pattern is similar and contacts are much stronger than in the head, which was used as control. These data show that even though e5 silencing may affect the robustness of the interactions, the spatial proximity between e5 and *Hoxa13* does not require the transcriptional activity of the enhancer. As *Hoxa13* expression is severely reduced in the absence of *Shh* [37], [41], we next wondered whether the contact pattern of *HoxA* genes with the distal limb enhancers was similarly preserved in *Shh−/−* limbs. To address this question, we compared the interaction profile of the *HoxA* cluster with its upstream regulatory region in wt and *Shh−/−* limbs, and in the head. For these 5C experiments, we used a modified 3C library protocol optimized for the production of libraries from a small number of cells (see Materials and Methods). This protocol largely recapitulated the contact pattern detected in wt limbs and the head with our standard approach (compare corresponding panels in Figures 2 and 4C). Consistent with our 3C data, this 5C analysis revealed a similar contact pattern between the 5′ *HoxA* genes and upstream regulatory region in the *Shh−/−* mutant and wt limbs (Figure 4C, Figure S3). These include contacts with e5 and e16 enhancers, which overlap with Gli3R sites and others like e10 and e13 that are not regulated by Shh. As observed in our preliminary 3C analysis, the contacts were weaker in the *Shh−/−* suggesting that strengthening a given enhancer-promoter contact upon enhancer activation may impact on the stabilization/strength of other interactions. Together, these data indicate that enhancer activity strengthens, but is not mandatory for spatial proximity between enhancers and their target genes.

### An extensive physical chromatin network regulates multiple genes in distal limb cells

**Figure 5:**
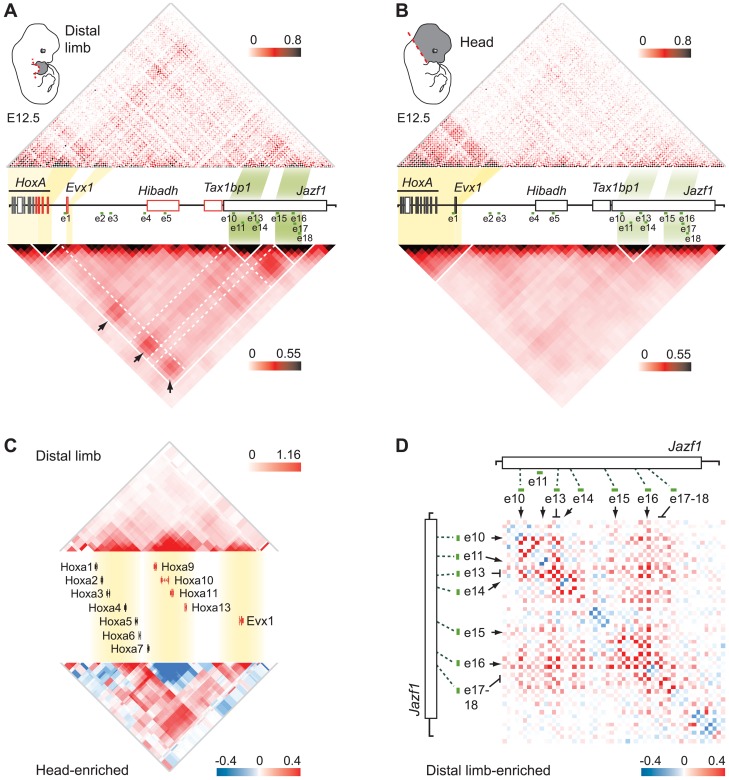
Extensive clustering of genes and enhancers highlights a complex regulation network in distal limbs. **A,B.** 5C interaction matrix of the *HoxA* cluster and its upstream regulatory region in distal limb (**A**) and head (**B**). The 5C data was generated by 5C-seq using tissues from E12.5 embryos, and is presented in the form of heatmaps according to color scales as described in Figure 2. Heatmaps above the linear diagram of the genomic region show interaction frequencies for each restriction fragment, irrespective of their size. Heatmaps at the bottom show the mean interaction frequencies per 20 kb DNA fragment and were obtained from binning and smoothing of the 5C raw data. Expressed genes within the region are colored in red. The yellow and green shading links the genomic position of *HoxA* and *Evx1* genes, and the enhancer clusters to the corresponding areas in heatmaps. Black arrows point to interactions between the gene sub-TADs and enhancer sub-TADs. White lines delineate the TAD and sub-TADs therein. Dashed white lines are drawn to highlight the sub-TAD interactions. **C.** Topological organization of the *HoxA* cluster and *Evx1*. Genes are organized in three sub-TADs in the limb (*top*). Interaction enrichment in head tissues compared to the limb (*bottom*) shows significant increase in interaction between the gene sub-TADs in the head. Smoothing was performed based on distance (8 kb) and heatmap intensities represent the mean of interaction frequency for each 8 kb window. **D.** Extensive limb-enriched interactions between distal *HoxA* enhancers suggest that a physical network regulates 5′ *HoxA* genes in the limb. The interaction matrix of the region containing enhancer e10 to e18 is shown in the form of a heatmap. Limb-enriched contacts are shown in red according to the color scale as described in Figure 2.

The observation that different enhancers drive transcription in the same areas of the limb suggests a possible physical link between some of them. To test this possibility, we extended our 5C analysis to the whole regulatory region. The *HoxA* cluster was previously found to span the junction between two adjacent TADs in human IMR90 and mouse embryonic stem cells (ES) analyzed with Hi-C at the mega-base scale [31]. We observed a similar megabase scale organization in our samples, where 5′ *HoxA* genes and distal limb enhancers are located in the same TAD (Figure 5A, B, and Figure S4). We found that this TAD is subdivided into domains of interactions that differ between the limb and head tissues at E12.5 (Figure 5A, B). In addition, contacts between sub-TADs are different in the two tissues. For example, the *HoxA* sub-TAD containing *Hoxa9* to *Hoxa13* preferentially forms long-range contacts with the enhancers in the limb (e.g. e10–14, e15–18; Figure 5A, Figure S5), while it interacts strongly with the 3′ *HoxA* genes in the head (Figure 5C, *bottom*, Figure S4). Similarly, *Evx1*, which spatially localizes within its own domain, interacts long-distance with a subset of the identified enhancers in limbs, consistent with its expression pattern being similar to *Hoxa13*. This is different in the head where *Evx1* and *HoxA* are mostly inactive and the genes form a large interacting domain (Figure 5B,C), which likely reflects chromatin compaction at transcriptionally silent loci. This result raises the possibility that chromatin conformation within TADs could vary in a tissue-specific manner. In support of this, a recent 5C analysis in mouse ES and neural progenitor cells identified tissue-specific topological domains at the sub-megabase scale, termed “sub-TADs” [33]. Our 5C analysis therefore revealed the existence of tissue-specific sub-TAD interactions underlying the regulation of *HoxA* genes in developing limbs.

The chromatin architecture resulting in the spatial proximity between 5′ *HoxA* genes, the enhancers, and the promoter of *Hibadh* suggests that *Hibadh* itself interacts with *HoxA*-associated limb enhancers. Indeed, *Hibadh* shows enriched interaction with e5, e13 and e16 in limb compared to head tissue (Figure S5). Interactions between *Evx1* and *Hibadh* are also enriched in limb compared to head tissue. Our experimental design unfortunately did not retain the promoter region of *Tax1bp1* and thus we could not profile its connectivity with the region. As for the promoter of *Jazf1*, it contacts neither the genes nor the enhancers consistent with the absence of RNAP2 and Med12 at its promoter (Figure 1D), and in agreement with previous work showing that *Jazf1* is expressed in distal limbs only at later developmental stages [16]. Together, these results show that a subset of *HoxA*-associated enhancers likely regulate also *Evx1* and *Hibadh*. Interestingly, there is an extensive connectivity between the enhancers themselves in the limb but not in head tissues (Figure 5A, B). Similarly to the genes, the enhancers partition among different sub-TADs that interact together. This is particularly visible for the most distal ones where e10–14 localized within one sub-TAD, and e15–18 into another (Figure 5A, D). This organization suggests that enhancers are spatially grouped into regulatory modules, which can interact with each other, eventually triggering specific expression patterns. Such interaction between genomic domains is reminiscent of the contacts identified in *Drosophila* [42]. It is thus likely that long-range gene regulation relies on sub-TAD interactions rather than discrete looping between specific DNA elements. Moreover, interactions between gene and enhancer sub-TADs in the limb strengthened and better defined the position of the corresponding TAD as compared to head tissues (Figure 5, compare A and B). This result suggests that although largely invariant, the partitioning of chromosomes into TADs can be affected by the tissue-specific folding of the chromatin at the sub-megabase level.

## Discussion

### Enhancer and gene sub-TADs interact long-range to control transcription in developing limbs

**Figure 6:**
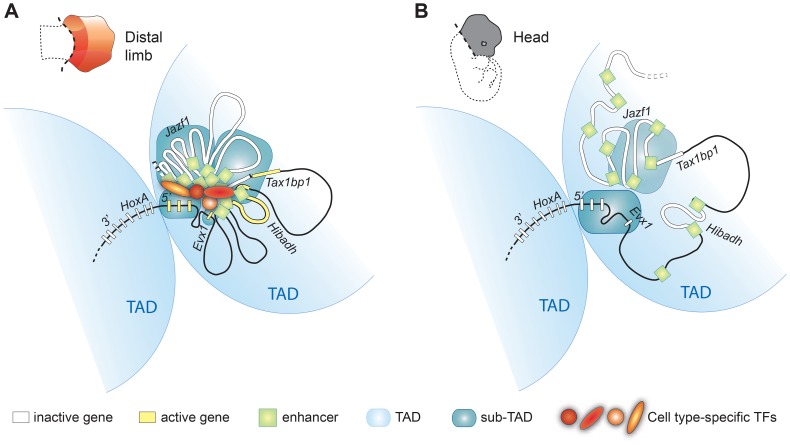
Model illustrating how genome topology underlies the tissue-specific regulation of *HoxA* genes. The *HoxA* cluster is partitioned between two TADs (light blue), physically segregating *3′HoxA* from 5′*HoxA* genes in a mostly cell-type independent manner. In contrast, the sub-TAD interaction pattern is drastically different in the limb (**A**) compared to the head (**B**). Limb enhancer sub-TADs (dark blue) interact with each other and with gene-sub-TADs in distal limb but not head tissue. Enhancer and gene interactions occur between sub-TADs from the same TAD (5′*HoxA* containing TAD) but not with 3′*HoxA* genes that are located in the adjacent TAD. The limb-specific sub-TAD interactions create a platform architecture controlling *HoxA* expression by the remote distal limb enhancers upon enhancer activation by transcription factors. The schemes of the chromatin conformation were designed assuming cellular homogeneity within each tissue.

In this study, we identified the very first set of *bona fide* limb enhancers controlling 5′ *HoxA* gene expression. We show that these enhancers, like the *HoxA* genes, are grouped into distinct topological domains at the sub-megabase scale, and that long-range contacts between the sub-TADs underlie the expression of 5′ *HoxA* genes in the developing limb. This result suggests that long-distance regulation of *HoxA* genes is based on sub-TAD interactions rather than discrete looping between enhancers and target genes. In the head, sub-TAD interactions are barely detectable thus indicating that the chromatin architecture of the region upstream *HoxA* varies in a tissue-specific manner at the sub-megabase scale. The apparent lack of sub-TAD interactions in the head could also be the consequence of the greater cellular complexity of this tissue, which would equally imply that sub-TAD interactions are cell type/tissue-specific (Figure 5B). A similar conclusion was recently reached based on the comparison between 5C data in mouse ES cells and neural progenitor cells {Nora, 2012 #165} [32]–[33]. The cell-type/tissue specificity of sub-TADs contrasts with the mostly invariant nature of TADs, which partition the genome into topological domains at the megabase scale [31]–[32]. While it was proposed that TADs could represent the structural basis of regulatory landscapes [43], the actual chromatin folding associated with transcriptional activity likely relies mostly on sub-TAD interactions (Figure 6). The diametrically opposed invariant nature of TADs and the tissue-specificity of sub-TADs also imply that distinct structural parameters define them. Accordingly, while arrays of CTCF sites characterize TAD boundaries [31], there is no obvious correlation between CTCF binding and the sub-TAD boundaries observed in limb buds (Figure S5).

Our results also indicate that at least some of the gene-enhancer contacts form independently of enhancer transcriptional activity (Figure 4), and we suggest that this structure largely exists before the gene transcription begins. This view is supported by the existence of interactions with loci for which there is no evidence of transcriptional activity (Blue stars in Figure 2). Moreover, our data shows that enhancers triggering distinct expression patterns in the limb (i.e. active in different cells) actually belong to the same sub-TAD, which further supports the notion that organization of the chromatin into sub-TADs does not simply reflect physical clustering of active enhancers. Chromatin interactions nonetheless appear strengthened by enhancer activity consistent with the recent concept of self-enforcing structure-function feedbacks, considered as a mechanism propagating cell-fate memory [44]. Our data also reveal better-defined boundaries of the 5′ *HoxA*-containing TAD in limbs, where sub-TADs robustly interact (Figure 5). This result raises the possibility that upon enhancer activation, the robustness of sub-TAD interactions within two adjacent TADs may change and consequently re-define the position of the TAD boundary. This potential TAD/sub-TAD interplay may actually provide a mechanistic explanation for *Hoxd9* to *Hoxd11* switching from one TAD to the neighboring one in proximal limb compared to distal limb cells [45].

The identification of multiple enhancers controlling 5′*HoxA* genes in distal limbs raises questions about the potential role and benefits for this apparently complex control mechanism. The evidence that the various enhancers identified have distinct spatial specificities, together with the eventual morphological diversity of the hand/foot, points to the existence of an early molecular heterogeneity among the mesenchymal progenitors of the hand/foot. Accordingly, Shh signaling regulates a subset of enhancers identified here while others are not (Figure 4). Nonetheless, most enhancers also appear to share overlapping functional domains. Interactions between some enhancers may reflect their co-function in some cells, which could correspond to specific cell populations in which a higher *HoxA* dosage is required. Alternatively, enhancer interactions could be the consequence of a “pre-set” chromatin architecture whereby a series of enhancers is brought in the vicinity of the same target genes, without having necessarily a combined transcriptional input in the same cell. Finally, it should also be mentioned that multiple enhancers with overlapping function can be beneficial, as exemplified with the discovery of shadow enhancers, which compensate for each another in sub-optimal conditions [46]–[47].

### Emergence of the hand and foot in Tetrapods is associated with the convergent evolution of *HoxA* and *HoxD* regulation

The hand/foot (autopod) is one of the major morphological novelties that accompanied Vertebrates adaptation to terrestrial habitats. As autopod development requires the function of *HoxA* and *HoxD* genes, the mechanism that led to the emergence of this new *Hox* function appears as a key molecular event associated with the fin-to-limb transition. By mapping active enhancers in the presumptive hand/foot and testing their interaction with *HoxA* genes, we provide evidence that *HoxA* expression in this tissue relies on long-range regulation by multiple enhancers. Previous studies on the regulation of *HoxD* genes led to the same conclusion [15], [48]–[49] suggesting that *HoxA* and *HoxD* genes have been recruited in this evolutionary novel structure through the implementation of a similar regulatory strategy. Yet, sequence comparison between *HoxA* and *HoxD* specific enhancers failed to identify obvious conservation thereby favoring a model whereby the recruitment of *HoxA* and *HoxD* genes in the presumptive hand/foot was likely achieved through independent implementations of novel *cis*-regulatory elements. Since these enhancers were identified with a resolution varying between 0.5 and 2 kb, it is possible that they are bound by the same transcription factors but with a distinct layout of their binding sites, as it would be expected from the independent evolution of the *HoxA* and *HoxD* regulatory landscapes. It is also possible that some ‘*HoxA*’ and ‘*HoxD*’ enhancers are bound by distinct combinations of transcription factors, in agreement with a subset of ‘*HoxA*’ enhancers having domains of activity within the developing limb distinct from the ‘*HoxD*’ enhancers (Figure 3; [15]). Notably, the differences in enhancer functional domains are consistent with the specificities of *HoxA* expression as illustrated in the presumptive digit one domain: while *HoxD* expression in digit one is restricted to *Hoxd13* as a result of the quantitative collinearity [50]–[52], no such phenomenon is observed for *HoxA* genes, the regulation of which involves a digit one-specific enhancer not identified for *HoxD* genes [15].

The independent evolution of *HoxA* and *HoxD* regulatory landscapes suggested by the absence of obvious sequence conservation of their respective enhancers is further supported by several findings. First, the recent evidence that *HoxA* and *HoxB* clusters most likely stem from the duplication of an ancestral *HoxA/B* cluster [53] implies that putative ancestral regulatory modules common only to *HoxA* and *HoxD* should have been lost at *HoxB* and *HoxC*. This scenario however appears unlikely to account for the specific *HoxA* and *HoxD* regulation associated with hand/feet development as the tandem duplications of the ancestral *Hox* cluster that led to the four Vertebrate *Hox* clusters occurred prior to the fin-to-limb transition. Second, there is a major difference in the layout of the *HoxA* and *HoxD* regulatory landscapes controlling their expression in the developing hand/feet. While *HoxD*-associated enhancers are part of a gene desert [15], [48]–[49], a large number of *HoxA*-associated enhancers are embedded in genes. Notably, *HoxA* enhancers with the highest enrichment of RNAP2, Med12 and p300, which also show the highest frequencies of interaction with *HoxA* genes, are located within *Hibadh* and *Jazf1*. Moreover, the genomic domain between *HoxA* and *Jazf1*, has undergone significant expansion from fish to mice (about 50 kb in fish and 800 kb in mice), indicating that an extensive genomic reshuffling at the *HoxA* regulatory landscape occurred during the fin-to-limb transition, which further support an independent evolution of the *HoxA* and *HoxD* regulatory landscapes. Interestingly, this genome expansion affected both the size of *Hibadh, Jazf1* and the intergenic regions. The absence of preferential localization of *HoxA*-associated enhancers in gene-free regions thus suggests that introns are equally amenable to sequence evolution and emergence of new regulatory elements.

Although enhancers controlling *HoxA* and *HoxD* expression in distal limb most likely emerged independently, in both cases the distal limb regulatory landscape is located within the TAD containing the 5′ genes ([45] and this work). As long-range physical contacts between DNA sequences preferentially occur within TADs, it is conceivable that topological constraints have influenced the evolution of *HoxA* and *HoxD* regulatory landscapes associated with their distal limb expression. Interestingly, the early/proximal limb regulatory landscape of *HoxD* was identified on the opposite side of the gene cluster, within a TAD containing the 3′ *HoxD* genes, and not contacting *Hoxd13* [45]. Whether the existence of a TAD boundary within the *HoxA* and *HoxD* clusters has favored the differential expression of *Hox* genes in proximal and distal limb bud or spatially constrained the emergence of proximal and distal limb enhancers remains unclear. Nonetheless, the deleterious modification of proximal limb development upon expression of *Hoxa13* or *Hoxd13* in early/proximal limb bud [54] raises the possibility that the TAD boundary embedded in both the *HoxA* and *HoxD* clusters has influenced the evolution of the tetrapod limb morphology. Although, chromosome partitioning into TADs remains to be characterized in most animal species, the presence of a TAD boundary embedded in each *Hox* cluster both in mice and humans [31] suggests a possible widespread impact of genome topology on the evolution of *Hox* regulation, and perhaps more generally on the evolution of regulatory landscapes.

In summary, our study reveals that extensive three-dimensional chromatin interactions control the expression of *HoxA* genes in developing limbs by forming distinct topological domains containing limb enhancers, which interact with each other and with the topological domains containing their target genes. Although this chromatin architecture is tissue-specific, our data suggests that it forms independently of enhancer activity, and is strengthened upon enhancer activation. Importantly, our data provide evidence that target genes and regulatory elements physically interact with each other through contacts between sub-TADs rather than by the formation of discreet “DNA loops”. From an evolutionary standpoint, the identification of *HoxA*-associated enhancers in limbs reveals major differences with the *HoxD* regulatory landscape suggesting that the changes in *HoxA* and *HoxD* regulation associated with the emergence of the hand/foot likely occurred through the independent emergence of regulatory sequences but common topological constraints.

## Materials and Methods

### Mice lines and transgenics

The *HoxA* ^Flox^, *HoxA* ^DelNeo^, IR50, and *Shh−/−* mice lines were described previously [1]–[2], [40]. Candidate enhancer sequences identified from ChIP-seq data were amplified by PCR using the primer sequences reported in Table S1 and PCR products were verified by sequencing. Enhancer sequences were cloned upstream of the chicken β-globin minimal promoter and the LacZΔCpG NLS reporter. Transgenic embryos were generated by pronuclear injections, and at least three transgenic embryos per construct were analyzed. The stable mouse line for e5 was generated using the same protocol.

### X-Gal staining and whole-mount in situ hybridization

X-Gal staining was performed on E12.5 embryos following standard procedures. *In situ* hybridization was conducted using a standard procedure [55]. Hygromycin and Neomycin probes were generated as described previously [2].

### Isolation and fixation of cells for ChIP-seq, 3C, and 5C analysis

Distal limb and head tissues were dissected at E12.5 for wt and at E11.5 for *Shh*−/− mice. Tissues were collected in 1×PBS containing 10% FBS (100 µl per embryo), and the samples were incubated 20 min at 37°C with collagenase (0.025% final concentration) to obtain single-cell suspensions. The number of cells in suspension was then counted under the microscope, and each sample was diluted in 9 ml of 1×PBS containing 10% FBS (5 ml for *Shh*−/− embryos). The cells were then fixed with 1% formaldehyde for 10 min at room temperature. Crosslinking was stopped with glycine (125 mM final concentration), and incubated 5 min at room temperature followed by 15 min on ice. Cells were centrifuged at 400 g for 10 min at 4°C. Supernatants were removed and the cell pellets were flash frozen on dry ice.

### Chromatin immunoprecipitation

Chromatin immunoprecipitation was performed as previously described with some modifications [56]–[57]. Briefly, chromatin from 5 million cells was sonicated using a Branson Sonicator 450D to obtain fragments with average sizes ranging between 100–600 bp. Cell debris was removed by centrifugation at 20,000 *g* for 15 min at 4°C and aliquots of the supernatant were taken for quantification and to confirm proper sonication. Remaining samples were stored at −80°C until use. Chromatin from 5 million cells was used for each immunoprecipitation. Protein G Dynal Beads (Invitrogen) were incubated 8 hours at 4°C with either 5 or 10 µg of antibodies. The chromatin was then incubated with the beads overnight. Immunoprecipitated complexes were sequentially washed in low salt (150 mM NaCl, 0.1% SDS, 1% Triton X-100, 2 mM EDTA, 20 mM Tris-HCl (pH 8.0)), medium salt (250 mM NaCl, 0.1% SDS, 1% Triton X-100, 2 mM EDTA, 20 mM Tris-HCl (pH 8.0)), LiCl (0.25 M LiCl, 0.5% NP40, 0.5% Na-Deoxycholate, 1 mM EDTA, 10 mM Tris-HCl (pH 8.0)), and 1×TE buffers. The protein/DNA complexes were eluted in an SDS buffer (1% SDS, 50 mM Tris (pH 8.0), 10 mM EDTA) by incubation at 65°C for 15 min on a rotating platform. Crosslinks were reversed by incubating the complexes at 65°C overnight. Samples were treated one hour at 55°C with RNAseA (0.2 µg/ml final concentration) and then with Proteinase K for two hours. Finally, the DNA was purified on QIAquick columns (Qiagen). Specific antibodies for Med12 and RNAP2 were purchased from Bethyl (A300-774A) and Abcam (ab5131), respectively.

### ChIP sequencing and analysis

The ChIPed material was sequenced on a Hi-Seq 2000 high-throughput DNA sequencer. Sequencing libraries were prepared from 31 ng (RNAP2), 5 ng (Med12), and 345 ng (input) of ChIPed DNA. The libraries and flow cells were prepared by the IRCM Molecular Biology and Functional Genomics platform. The libraries were multiplexed and sequenced on one lane. The sequencing was performed by the McGill University and Génome Québec Innovation Centre following recommendations by the manufacturer (Illumina, San Diego, CA).

For RNAP2, Med12, and the input, we obtained a total of 151,045,509, 110,507,927, and 98,043,425 sequence reads, respectively. The first base of each sequence read was trimmed to ensure high base calling quality. The trimmed reads were mapped to the mouse mm9 genome assembly with Bowtie using the –best parameters [58]. To identify the highly significant RNAP2 and Med12 peaks, we used the MACS 1.4.1 peak finder with the following parameters: --format SAM --wig --bw 250 --mfold 7,30 -pvalue 1e–5 -g mm [59].

Redundant reads were filtered out for peak finding and wiggle file generation. Thus the wiggle files enclose the total number of uniquely mapped and non-redundant reads. After processing the data, the number of sequence reads we obtained was 129,222,085 for RNAP2, 91,816,355 for Med12, and 88,141,136 for the input. The position of RNAP2 and Med12 peaks genome-wide identified in our study is provided in Tables S21 and S22, respectively. We also provide the wig files for the data on chromosome 6 (Dataset S1, S2 and S3).

### 3C library preparation for a large number of cells (2×10^6^–10^7^ cells)

Limb and head cell pellets were treated as previously described [29], [60]. Briefly, 10 million fixed cells (2.87 million for *Shh*−/− library used for the 3C experiments) were incubated for 15 min on ice in 200 µl of lysis buffer (10 mM Tris (pH 8.0), 10 mM NaCl, 0.2% NP40, supplemented with fresh protease inhibitor cocktail). Cells were then disrupted on ice with a dounce homogenizer (pestle B; 2×20 strokes). Cell suspensions were transferred to eppendorf tubes and centrifuged 5 min at 2000 g. Supernatants were removed and the cell pellets were washed twice with 100 µl of 1×EcoRI buffer (NEB). After the second wash, the cell pellet was resuspended in 100 µl of 1×EcoRI buffer, and divided into two eppendorf tubes containing 50 µl of cell suspension. 1×EcoRI buffer (337 µl) was added to each tube, and the mixture was incubated 10 min at 65°C with 0.1% SDS final (38 µl). Triton X-100 (44 µl of 10% Triton X-100) was added before overnight digestion with EcoRI (400 Units). The restriction enzyme was then inactivated by adding 86 µl of 10% SDS, and incubating 30 min at 65°C. Samples were then individually diluted into 7.62 ml of ligation mix (750 µl of 10% Triton X-100, 750 µl of 10×ligation buffer, 80 µl of 10 mg/ml of BSA, 80 µl of 100 mM ATP and 3000 Cohesive end Units of T4 DNA ligase). Ligation was carried out at 16°C for 2 hours.

3C libraries were then incubated overnight at 65°C with 50 µl Proteinase K (10 mg/ml), and with an additional 50 µl Proteinase K the following day for 2 hours. The DNA was purified by one phenol and one phenol-chloroform extraction, and precipitated with 0.1 volume of 3M NaOAc pH 5.2 (800 µl) and 2.5 volumes of cold EtOH (20 ml). After at least 1 h at −80°C, the DNA was centrifuged 25 min at 20,000 g at 4°C, and the pellets were washed with cold 70% EtOH. The DNA was resuspended in 400 µl of 1×TE pH 8.0, and transferred to eppendorf tubes for another phenol-chloroform extraction and precipitation with 0.1 volume of 3M NaOAc pH 5.2 (40 µl) and 2.5 volumes of cold EtOH (1.1 ml). DNA was recovered by centrifugation (25 min at maximum speed at 4°C), and washed eight times with cold 70%EtOH. The pellets were then dissolved in 100 µl of 1×TE pH 8.0, and incubated with RNAse A (1 µl at 10 mg/ml) for 15 min at 37°C.

### 3C library preparation for a small number of cells (10^6^ cells)

This protocol was used to produce the 5C data for the distal limb, *Shh−/−* distal limb, and head shown in Figure 4. The protocol is essentially the same as the one described for samples containing 2 to 10 million cells, with some modifications. Briefly, one million fixed cells were incubated for 15 min on ice in 200 µl of lysis buffer (10 mM Tris (pH 8.0), 10 mM NaCl, 0.2% NP40 supplemented with fresh protease inhibitor cocktail). Cells were then disrupted on ice with a dounce homogenizer (pestle B; 2×20 strokes). Cell suspensions were transferred to eppendorf tubes and centrifuged 5 min at 2000 g. Supernatants were removed and the cell pellets were washed twice with 100 µl of 1×EcoRI buffer (NEB).

After the second wash, the cell pellet was resuspended in 50 µl of 1×EcoRI buffer. 1×EcoRI buffer (337 µl) was added to each tube, and the mixture was incubated 10 min at 65°C with 0.1% SDS final (38 µl). Triton X-100 (44 µl of 10% Triton X-100) was added before overnight digestion with EcoRI (400 Units). The restriction enzyme was then inactivated by incubating 30 min at 65°C. Ligation was performed in 600 µl (450 µl of digestion product, 15 µl of 10% Triton-X-100, 60 µl of ligase buffer, 6 µl of 10 mg/ml of BSA, 6 µl of 10 mM ATP, and 300 Cohesive end Units of T4 DNA ligase). Ligation was carried out at 16°C for 4 hours.

3C libraries were then incubated overnight at 65°C with 15 µl Proteinase K (10 mg/ml), and with an additional 15 µl Proteinase K the following day for 2 hours. The DNA was purified by one phenol and two phenol-chloroform extractions, and precipitated with 0.1 volume of 3M NaOAc pH 5.2 (64 µl) and 2.5 volumes of cold EtOH (1740 µl). After at least 1 h at −80°C, the DNA was centrifuged 25 min at maximum speed at 4°C, and the pellets were washed once with cold 70% EtOH. The DNA was resuspended in 50 µl of 1×TE pH 8.0, and incubated with RNAse A (1 µl at 10 mg/ml) for 15 min at 37°C.

### Design and preparation of control 3C libraries

As 3C products are quantified by PCR amplification of expected ligation junctions with different primer pairs, differences in PCR primer pair efficiencies must be corrected using control 3C libraries. Control libraries were generated from bacterial artificial chromosomes (BACs) as previously described [29] and contain equimolar ratios of all possible 3C contacts. Briefly, BAC clones covering the *HoxA* region (mm9, chr6: 51,946,668–52,656,241), and one *USP22* control region (mm9, chr11: 60,890,403–61,093,236)) were mixed in equimolar ratio. Mixed BACs were digested with EcoRI and randomly ligated with T4 DNA ligase (5700 Cohesive end Units) overnight at 16°C. BAC libraries were then purified by phenol-chloroform extraction. The libraries were generated with the following BACs: RP23-420L19, RP24-359H1, RP24-242G11, RP-347D14, RP23-305I5 (Invitrogen, CHORI). These libraries were used only to correct primer pair efficiencies during 3C analysis and not in the 5C experiments.

### 3C analysis

3C primers were designed using the ‘3CPrimer’ program (http://dostielab.biochem.mcgill.ca), and sequences are listed in Table S2. Three reactions using the control BAC library and three reactions using each 3C library were generated for each primer pair. The PCR conditions were described elsewhere [29]. 3C PCR products were resolved on agarose gel containing ethidium bromide and quantified using a ChemiDoc XRS system featuring a 12-bit digital camera and the Quantity One computer software (version 4.6.3; BioRad). Interaction frequencies (IF) were measured by dividing the value of each template PCR reactions by the value of each of the three control PCR reactions. The nine values were then average to determine the normalized interaction frequency. Three biological replicates were averaged after normalization for the wt limb and head. Normalization between different libraries was done using the compaction profiles for the *USP22* region and an intergenic region within *HoxA* region as a reference.

### 5C primer and library design

5C primers covering the *USP22* region (mm9, chr11: 60,917,307–61,017,307) and the *HoxA* region (mm9, chr6: 52,099,908–53,050,000) were designed using ‘my5C.primer’ [61] and the following parameters: optimal primer length of 30 nt, optimal TM of 65°C, default primer quality parameters (mer:800, U-blast:3, S-blasr:50). The sequences of these primers are listed in Table S3 and S4. Primers were not designed for large (>20 kb) and small (<100 bp) restriction fragments. Low complexity and repetitive sequences were excluded from our experimental designs such that not all fragments could be probed in our assays. Primers with several genomic targets were also removed.

The universal A-key (CCATCTCATCCCTGCGTGTCTCCGACTCAG-(5C-specific)) and the P1-key tails ((5C-specific)-ATCACCGACTGCCCATAGAGAGG) were added to the Forward and Reverse 5C primers, respectively. Reverse 5C primers were phosphorylated at their 5′ ends. Two experimental designs were used in our study. In the “cluster R” design (anchored 5C scheme, Figure 2, Figure S1, Figure 4C, Figure S3), Reverse 5C primers covered the *HoxA* cluster while Forward 5C primers tiled the surrounding upstream region. In this design, we used 142 Forward and 39 Reverse 5C primers (133 Forward/30 Reverse for the *HoxA* region, 9 Forward/9 Reverse *USP22* region). In the “FR” design (alternating 5C scheme, Figure 5, Figure S3), alternating Forward and Reverse 5C primers covering the entire *HoxA* region were used to generate the 5C libraries. This design used 194 primers (86 Forward/90 Reverse for the *HoxA* region, 9 Forward/9 Reverse *USP22* region). Primer sequences are listed in Table S3 (anchored “R” design) and S4 (alternating “FR” design).

### 5C library preparation

5C libraries were prepared and amplified with the A-key and P1-key primers following a procedure described previously [30]. Briefly, 3C libraries were first titrated by PCR for quality control (single band, absence of primer dimers, etc.), and to verify that contacts were amplified at frequencies similar to what is usually obtained from comparable libraries (same DNA amount from the same species and karyotype) [29], [62]–[63]. We also verified the quality of the 3C libraries by generating a compaction profile in the *USP22* region. In general, we used approximately 1.5 µg of 3C library per 5C ligation reaction when the libraries were generated from a large number of cells (2×10^6^ to 10^7^ cells). When 3C libraries were generated from a small cell number (10^6^ cells), we used approximately 1 µg of DNA.

Before adding the 3C libraries to the reaction tubes, 5C primer stocks (20 µM) were diluted individually in water on ice, and mixed to a final concentration of 0.002 µM. Mixed diluted primers (1.7 µl) were combined with 1 µl of annealing buffer (10×NEBuffer 4, New England Biolabs Inc.) on ice in reaction tubes. Salmon testis DNA (1.5 µg) was added to each 5C reaction, followed by the 3C libraries and water for a final volume of 10 µl. Samples were denatured at 95°C for 5 min, and annealed at 55°C for 16 hours. Ligation with Taq DNA ligase (10 U) was performed at 55°C for one hour. One tenth (3 µl) of each ligation was then PCR-amplified individually with primers against the A-key and P1-key primer tails. We used 28 cycles based on dilution series showing linear PCR amplification within that cycle range. The products from 2 (for the 3C libraries prepared from a large number of cells) to 8 (for the 3C libraries prepared from 10^6^ cells) PCR reactions were pooled before purifying the DNA on MinElute columns (Qiagen).

5C libraries were quantified on agarose gel and diluted to 0.0534 ng/µl (for Xpress Template Kit v2.0) or 0.0216 ng/µl (for Ion PGM Template OT2 200 kit). One microliter of diluted 5C library was used for sequencing with an Ion PGM Sequencer. Samples were sequenced onto Ion 316 Chips following either the Ion Xpress Template Kit v2.0, and Ion Sequencing Kit v2.0 protocols, or the Ion PGM Template OT2 200 Kit, and Ion PGM Sequencing 200 Kit v2.0 protocols as recommended by the manufacturer's instructions (Life Technologies).

### 5C analysis

Analysis of the 5C sequencing data was performed as described earlier [30]. The sequencing data was processed through a Torrent 5C data transformation pipeline on Galaxy (https://main.g2.bx.psu.edu/). Briefly, the data was mapped against a customized reference file with TMAP. The reference file contained a list of all possible contacts between Forward and Reverse 5C primers covering our regions. The data was then filtered to remove low-quality reads (MAQ quality score of lower than 30), reads aligning more than two nucleotides away from the reference sequence start site, and reads which do not contain EcoRI restriction sites. This analysis generates an excel sheet containing interaction frequency lists (IFL) as well as a text file, which was used to visualize results using ‘my5C-heatmap’ [61]. Limb-enriched 5C interactions were obtained by subtracting limb and head 5C-seq data. Data was normalized by dividing the number of reads of each 5C contact by the total number of reads from the corresponding sequence run. All scales correspond to this ratio multiplied by 10^3^. The number of total reads and of used reads is provided for each experiment in Table S5. 5C data are provided in Tables S6 to S20 and can be downloaded from our website: http://dostielab.biochem.mcgill.ca/

### Databases and URLs

The limb p300 and H3K27Ac datasets (Acc. No. GSE13845 and GSE30641) are from E11.5 embryos, and were downloaded from the Gene Expression Omnibus (GEO) website http://www.ncbi.nlm.nih.gov/geo/. The my5C-primer and my5C-heatmap bioinformatics tools can be found at http://3dg.umassmed.edu/my5Cheatmap/heatmap.php

## Supporting Information

Dataset S1 ChIP RNAP2 chr6 wig file. A compressed wig file containing the ChIP-seq results of RNAP2 in E12.5 mouse distal limb from chromosome 6. This file can be uploaded directly onto the UCSC genome browser (http://genome.ucsc.edu/) after decompression.
(GZ) Click here for additional data file.

Dataset S2 ChIP Med12 chr6 wig file. A compressed wig file containing the ChIP-seq results of Med12 in E12.5 mouse distal limb from chromosome 6. This file can be uploaded directly onto the UCSC genome browser (http://genome.ucsc.edu/) after decompression.
(GZ) Click here for additional data file.

Dataset S3 ChIP input chr6 wig file. A compressed wig file containing the sequencing data of the input from E12.5 mouse distal limb on chromosome 6. This file can be uploaded directly onto the UCSC genome browser (http://genome.ucsc.edu/) after decompression.
(GZ) Click here for additional data file.

Figure S1 Interactions between candidate enhancers and 5′ *HoxA* genes in the limb are reproduced in biological replicates. Physical contacts between the *HoxA* cluster and the upstream genomic region containing candidate enhancers were measured by 5C-seq in two biological replicates of distal limb (*top, middle*), and a biological replicate of the head (*bottom*) of E12.5 embryos. The color intensity of each pixel in 5C heatmaps reflects the frequency of interaction between two genomic regions. Contact frequency is according to the respective color scales and corresponds to the number of sequence reads. Most predicted enhancers (4;5;10;13;14;15;16;17;18) interacted long-distance specifically with 5′ *HoxA* genes in the limb and contacts were weaker or absent in the head (compare top two panels with bottom). Green dotted lines link the position of enhancers along the genomic region to corresponding 5C fragments in the heatmaps. Brackets on the left hand side of each heatmap show the area containing *Hoxa9*, *a10*, *a11*, and *a13*. Green arrows indicate the chromatin fragments containing the *Hibadh* and *Jazf1* promoters (p). Other limb-enriched interactions with *HoxA* genes that do not correspond to candidate enhancers are highlighted by blue stars. Restriction fragments corresponding to enhancer e6–8, 12, and 19 could not be included in the 5C design as they fell into regions that were not amenable to 5C (see Materials and Methods).
(EPS)

Figure S2 Interaction of the e2–e5 enhancers with *Hoxa13* is more frequent in the limb than in the head. Physical contacts between the enhancers and the *Hoxa13* promoter were detected by 3C as described in the Materials and Methods. The position of the enhancers along the genomic region is indicated below the linear diagram and is highlighted in green. Active genes are shown in red. Each contact was measured at least three times from both the tissue and control libraries. Error bars represent the standard error of the mean.
(EPS)

Figure S3 Biological replicates confirm the interactions between candidate enhancers and 5′ *HoxA* genes in *Shh−/−* mutant limbs. Physical contacts between the *HoxA* cluster and the upstream genomic region containing the limb enhancers were measured by 5C-seq in two biological replicates of E11.5 *Shh−/−* distal limb (*top, middle*), and a biological replicate of E12.5 head (*bottom*). The color intensity of each pixel in 5C heatmaps reflects the frequency of interaction between two genomic regions. Contact frequency is according to the respective color scales and corresponds to the number of sequence reads. The limb-specific interaction pattern between enhancers and the 5′ *HoxA* genes are similar in *Shh−/−* (*middle* panel) and wt distal limb buds (*top* panel) albeit with some interaction frequencies slightly reduced. These data are consistent with the data shown in Figure 4. Dotted lines delineate the regions containing the enhancers bound by Gli3R (e3, e5 and e16). Brackets on the left hand side of each heatmap shows the area containing *Hoxa9*, *a10*, *a11*, and *a13*. Green arrows indicate the chromatin fragments containing the *Hibadh* and *Jazf1* promoters (p). Restriction fragments corresponding to enhancer e6–8, 12, and 19 could not be included in the 5C design as they fell into regions that were not amenable to 5C (see Materials and Methods).
(EPS)

Figure S4 The spatial organization of the *HoxA* regulatory region at the megabase and sub-megabase scale in limb and head tissues. 5C interaction matrix of the *HoxA* cluster and its upstream regulatory region in distal limb (**A**) and head tissue (**B**). The 5C data was generated by 5C-seq using tissues from E12.5 embryos, and is presented in the form of heatmaps according to color scales as described in Figure 2. Heatmaps above the linear diagram of the genomic region show interaction frequencies for each restriction fragment, irrespective of their size. Heatmaps at the bottom show the mean interaction frequencies per 20 kb DNA fragment and were obtained from binning and smoothing of the 5C raw data. Black arrows point to interactions between the gene sub-TADs and enhancer sub-TADs. White lines delineate the TAD and sub-TADs therein, and dashed white lines are drawn to highlight the sub-TAD interactions. Expressed genes are shown in red. The yellow and green shading links the genomic position of *HoxA* and *Evx1* genes, and the enhancer clusters to the corresponding areas in heatmaps.
(EPS)

Figure S5 Comparison between interaction patterns in the limb and head tissues shows major changes in chromatin architecture at the sub-megabase level. The interaction matrix of the region containing enhancer e1 to e5 with the entire distal limb regulatory landscape is shown in heatmap form. Interactions were measured by 5C-seq. The color scale represents differences in interaction frequencies in the head and limb. The robust interactions between sub-TADs in limb (black circles) modify the internal TAD architecture. Dashed circles highlight the enriched interaction between the region containing e5 and the *Hibadh* promoter, and the enhancer sub-TADs. Enriched interaction between *Evx1* and a subset of distal limb enhancers are shown with grey circles. Upon sub-TAD interactions in limbs, some loci get pulled away (blue) from each other compared to the head while other become closer (pink). The most enriched interactions not involving an enhancer or promoter could also represent structural contacts as those in Figure 2. Binding sites of cohesin and CTCF identified by ChIP-seq in E11.5 limb [35] are indicated by grey and black bars, respectively.
(EPS)

Table S1 Results of transgenesis. Summary of the information relevant to the genomic regions tested for enhancer activity by transgenesis shown in Figure 3.
(XLS)

Table S2 Mouse 3C primers for the *HoxA*, enhancer, and *USP22* regions. The 3C primer sequences used to characterize the *HoxA* cluster and its regulatory landscape, and the genomic region containing housekeeping *USP22* gene are listed along with their genomic position and the corresponding 3C fragment tested.
(XLSX)

Table S3 Mouse 5C primers for *HoxA* and *USP22* regions used in the anchored “R” design. This 5C primer set was used in Figures 2, 4, S1, and S3.
(XLSX)

Table S4 Mouse 5C primers for *HoxA* and *USP22* regions used in the altered “FR” design. This 5C primer set was used in Figures 5, S4, and S5.
(XLSX)

Table S5 5C sequencing results. Summary of the number of sequence reads before and after processing from each 5C dataset presented in this study.
(XLSX)

Table S6 Mouse 5C results for the distal limb anchored “cluster R” design. Distal limb 5C dataset from Figure 2 (*top*) presented in matrix format. Predicted EcoRI restriction fragments and corresponding genomic regions are named at the top of each column and on the left of each row. Intersecting rows and columns identify the pair-wise contacts corresponding to interaction frequencies. The 5C data was filtered as described in Materials and Methods, and is normalized based on the number of sequence reads per 1,000.
(XLSX)

Table S7 Mouse 5C results for the distal limb replicate 1 anchored “cluster R” design. First biological replicate of the distal limb 5C dataset presented in matrix format. This data is shown in Figure S1 (*top*). Predicted EcoRI restriction fragments and corresponding genomic regions are named at the top of each column and on the left of each row. Intersecting rows and columns identify the pair-wise contacts corresponding to interaction frequencies. The 5C data was filtered as described in Materials and Methods, and is normalized based on the number of sequence reads per 1,000.
(XLSX)

Table S8 Mouse 5C results for the distal limb replicate 2 anchored “cluster R” design. Second biological replicate of the distal limb 5C dataset presented in matrix format. This data is shown in Figure S1 (*middle*). Predicted EcoRI restriction fragments and corresponding genomic regions are named at the top of each column and on the left of each row. Intersecting rows and columns identify the pair-wise contacts corresponding to interaction frequencies. The 5C data was filtered as described in Materials and Methods, and is normalized based on the number of sequence reads per 1,000.
(XLSX)

Table S9 Mouse 5C results for the head anchored “cluster R” design. Head 5C dataset from Figure 2 (*middle*) presented in matrix format. Predicted EcoRI restriction fragments and corresponding genomic regions are named at the top of each column and on the left of each row. Intersecting rows and columns identify the pair-wise contacts corresponding to interaction frequencies. The 5C data was filtered as described in Materials and Methods, and is normalized based on the number of sequence reads per 1,000.
(XLSX)

Table S10 Mouse 5C results for the head replicate 1 anchored “cluster R” design. Biological replicate of the Head 5C dataset presented in matrix format. This data is shown in Figure S1 (*bottom*). Predicted EcoRI restriction fragments and corresponding genomic regions are named at the top of each column and on the left of each row. Intersecting rows and columns identify the pair-wise contacts corresponding to interaction frequencies. The 5C data was filtered as described in Materials and Methods, and is normalized based on the number of sequence reads per 1,000.
(XLSX)

Table S11 Mouse 5C results for the distal limb alternating “FR” design. Distal limb 5C dataset from Figure 5A presented in matrix format. Predicted EcoRI restriction fragments and corresponding genomic regions are named at the top of each column and on the left of each row. Intersecting rows and columns identify the pair-wise contacts corresponding to interaction frequencies. The 5C data was filtered as described in Materials and Methods, and is normalized based on the number of sequence reads per 1,000. This dataset was used to generate Figures 5C, 5D and S5.
(XLSX)

Table S12 Mouse 5C results for the distal limb replicate alternating “FR” design. Biological replicate of the distal limb 5C dataset presented in matrix format. This data is shown in Figure S4A. Predicted EcoRI restriction fragments and corresponding genomic regions are named at the top of each column and on the left of each row. Intersecting rows and columns identify the pair-wise contacts corresponding to interaction frequencies. The 5C data was filtered as described in Materials and Methods, and is normalized based on the number of sequence reads per 1,000.
(XLSX)

Table S13 Mouse 5C results for the head alternating “FR” design. Head 5C dataset from Figure 5B presented in matrix format. Predicted EcoRI restriction fragments and corresponding genomic regions are named at the top of each column and on the left of each row. Intersecting rows and columns identify the pair-wise contacts corresponding to interaction frequencies. The 5C data was filtered as described in Materials and Methods, and is normalized based on the number of sequence reads per 1,000. This dataset was used to generate Figures 5C, 5D and S5.
(XLSX)

Table S14 Mouse 5C results for the head replicate alternating “FR” design. Biological replicate of the head 5C dataset presented in matrix format. This data is shown in Figure S4B. Predicted EcoRI restriction fragments and corresponding genomic regions are named at the top of each column and on the left of each row. Intersecting rows and columns identify the pair-wise contacts corresponding to interaction frequencies. The 5C data was filtered as described in Materials and Methods, and is normalized based on the number of sequence reads per 1,000.
(XLSX)

Table S15 Mouse 5C results for the distal limb anchored “cluster R” design (using the 3C library protocol for a small number of cells). Distal limb 5C dataset from Figure 4C (*top*) presented in matrix format. This 5C data was generated from wt distal limbs 3C libraries produced with a 3C protocol for a small number of cells (see Materials and Methods). Predicted EcoRI restriction fragments and corresponding genomic regions are named at the top of each column and on the left of each row. Intersecting rows and columns identify the pair-wise contacts corresponding to interaction frequencies. The 5C data was filtered as described in Materials and Methods, and is normalized based on the number of sequence reads per 1,000.
(XLSX)

Table S16 Mouse 5C results for the distal limb of *Shh−/−* anchored “cluster R” design (using the 3C library protocol for a small number of cells). *Shh−/−* distal limb 5C dataset from Figure 4C (*middle*) presented in matrix format. This 5C data was generated from *Shh−/−* distal limbs 3C libraries produced with a 3C protocol for a small number of cells (see Materials and Methods). Predicted EcoRI restriction fragments and corresponding genomic regions are named at the top of each column and on the left of each row. Intersecting rows and columns identify the pair-wise contacts corresponding to interaction frequencies. The 5C data was filtered as described in Materials and Methods, and is normalized based on the number of sequence reads per 1,000.
(XLSX)

Table S17 Mouse 5C results for the distal limb of *Shh−/−* replicate 1 anchored “cluster R” design (using the 3C library protocol for a small number of cells). First biological replicate of the *Shh−/−* distal limb 5C dataset presented in matrix format. This 5C data is shown in Figure S3 (*top*), and was generated from *Shh−/−* distal limbs 3C libraries produced with a 3C protocol for a small number of cells (see Materials and Methods). Predicted EcoRI restriction fragments and corresponding genomic regions are named at the top of each column and on the left of each row. Intersecting rows and columns identify the pair-wise contacts corresponding to interaction frequencies. The 5C data was filtered as described in Materials and Methods, and is normalized based on the number of sequence reads per 1,000.
(XLSX)

Table S18 Mouse 5C results for the distal limb of *Shh−/−* replicate 2 anchored “cluster R” design (using the 3C library protocol for a small number of cells). Second biological replicate of the *Shh−/−* distal limb 5C dataset presented in matrix format. This 5C data is shown in Figure S3 (*middle*), and was generated from *Shh−/−* distal limbs 3C libraries produced with a 3C protocol for a small number of cells (see Materials and Methods). Predicted EcoRI restriction fragments and corresponding genomic regions are named at the top of each column and on the left of each row. Intersecting rows and columns identify the pair-wise contacts corresponding to interaction frequencies. The 5C data was filtered as described in Materials and Methods, and is normalized based on the number of sequence reads per 1,000.
(XLSX)

Table S19 Mouse 5C results for the head anchored “cluster R” design (using the 3C library protocol for a small number of cells). Head 5C dataset from Figure 4C (*bottom*) presented in matrix format. This 5C data was generated from wt head tissue 3C libraries produced with a 3C protocol for a small number of cells (see Materials and Methods). Predicted EcoRI restriction fragments and corresponding genomic regions are named at the top of each column and on the left of each row. Intersecting rows and columns identify the pair-wise contacts corresponding to interaction frequencies. The 5C data was filtered as described in Materials and Methods, and is normalized based on the number of sequence reads per 1,000.
(XLSX)

Table S20 Mouse 5C results for the head replicate 1 anchored “cluster R” design (using the 3C library protocol for a small number of cells). Biological replicate of the head 5C dataset presented in matrix format. This 5C data is shown in Figure S3 (*bottom*), and was generated from head tissue 3C libraries produced with a 3C protocol for a small number of cells (see Materials and Methods). Predicted EcoRI restriction fragments and corresponding genomic regions are named at the top of each column and on the left of each row. Intersecting rows and columns identify the pair-wise contacts corresponding to interaction frequencies. The 5C data was filtered as described in Materials and Methods, and is normalized based on the number of sequence reads per 1,000.
(XLSX)

Table S21 RNAP2 peaks in E12.5 mouse distal limb. The start and end position of the RNAP2 peaks identified in mouse distal limbs at E12.5 is listed genome-wide.
(XLSX)

Table S22 Med12 peaks in E12.5 mouse distal limb. The start and end position of the Med12 peaks identified in mouse distal limbs at E12.5 is listed genome-wide.
(XLSX)
